# 5,5′-Dimeth­oxy-2,2′-[(nonane-1,9-diyldi­oxy)bis­(nitrilo­methyl­idyne)]diphenol

**DOI:** 10.1107/S1600536809041038

**Published:** 2009-10-17

**Authors:** Li Li, Hong-Zheng Ma, Su-Xia Gao, Wen-Kui Dong, Jian-Chao Wu

**Affiliations:** a School of Chemical and Biological Engineering, Lanzhou Jiaotong University, Lanzhou 730070, People’s Republic of China; b The Five TBM, China Railway Tunnel Co. Ltd, Xinxiang 453000, People’s Republic of China; c School of Environmental Science and Municipal Engineering, Lanzhou Jiaotong University, Lanzhou 730070, People’s Republic of China

## Abstract

The mol­ecule of the title compound, C_25_H_34_N_2_O_6_, adopts a fully extended configuration. The oxime (—CH=N—O—) group is coplanar with the aromatic ring and the two benzene rings are almost parallel, making a dihedral angle of 0.16 (3)°. In the crystal structure, strong intra­molecular O—H⋯N hydrogen bonds generate six-membered *S*(6) ring motifs. Inter­molecular C—H⋯O hydrogen bonds link each mol­ecule to five others, forming an infinite three-dimensional supra­molecular structure. The crystal is further stabilized by π–π stacking inter­actions between neighbouring benzene rings [centroid–centroid distance = 3.744 (2) Å].

## Related literature

For related literature, see: Bernstein *et al.* (1995 ▶); Campbell *et al.* (2001 ▶); Desiraju (1996 ▶); Dong, He, Guan *et al.* (2008 ▶); Dong, He, Li *et al.* (2008 ▶); Dong, Li *et al.* (2008 ▶); Dong *et al.* (2009 ▶); Mohand *et al.* (1995 ▶); Sun *et al.* (2009 ▶).
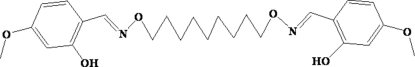


## Experimental

### 

#### Crystal data

C_25_H_34_N_2_O_6_
*M* *_r_* = 458.54
Triclinic, 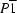

*a* = 10.4701 (10) Å
*b* = 11.1249 (12) Å
*c* = 12.7602 (14) Å
α = 65.544 (1)°
β = 86.664 (2)°
γ = 67.085 (1)°
*V* = 1236.7 (2) Å^3^
*Z* = 2
Mo *K*α radiation
μ = 0.09 mm^−1^
*T* = 298 K
0.48 × 0.46 × 0.21 mm

#### Data collection

Bruker SMART 1000 CCD area-detector diffractometer
Absorption correction: multi-scan (*SADABS*; Sheldrick, 1996 ▶) *T*_min_ = 0.959, *T*_max_ = 0.982
6463 measured reflections
4303 independent reflections
2050 reflections with *I* > 2σ(*I*)
*R*_int_ = 0.036

#### Refinement

*R*[*F* ^2^ > 2σ(*F* ^2^)] = 0.049
*wR*(*F* ^2^) = 0.125
*S* = 1.05
4303 reflections
300 parameters
H-atom parameters constrained
Δρ_max_ = 0.16 e Å^−3^
Δρ_min_ = −0.18 e Å^−3^

### 

Data collection: *SMART* (Siemens, 1996 ▶); cell refinement: *SMART*; data reduction: *SAINT* (Siemens, 1996 ▶); program(s) used to solve structure: *SHELXS97* (Sheldrick, 2008 ▶); program(s) used to refine structure: *SHELXL97* (Sheldrick, 2008 ▶); molecular graphics: *SHELXTL* (Sheldrick, 2008 ▶); software used to prepare material for publication: *SHELXTL*.

## Supplementary Material

Crystal structure: contains datablocks global, I. DOI: 10.1107/S1600536809041038/hg2576sup1.cif

Structure factors: contains datablocks I. DOI: 10.1107/S1600536809041038/hg2576Isup2.hkl

Additional supplementary materials:  crystallographic information; 3D view; checkCIF report

## Figures and Tables

**Table 1:** Hydrogen-bond geometry (Å, °)

*D*—H⋯*A*	*D*—H	H⋯*A*	*D*⋯*A*	*D*—H⋯*A*
C17—H17*B*⋯O4^i^	0.96	2.68	3.369 (3)	129
C18—H18⋯O3^ii^	0.93	2.40	3.295 (3)	160
C25—H25*C*⋯O3^iii^	0.96	2.59	3.541 (4)	170
C25—H25*B*⋯O6^iv^	0.96	2.61	3.469 (4)	150
O3—H3⋯N1	0.82	1.86	2.583 (4)	147
O5—H5⋯N2	0.82	1.94	2.663 (4)	147

Symmetry codes: (i) 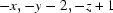; (ii) 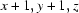; (iii) 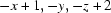; (iv) 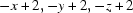.

**Fig. 1.**
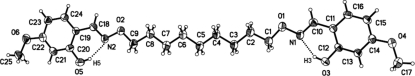
The molecular structure of the title compound with atom numbering scheme. Displacement ellipsoids for non-hydrogen atoms are drawn at the 30% probability level.

**Fig. 2.**
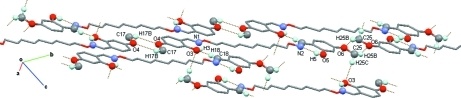
Part of the supramolecular structure of the title compound. Intra- and intermolecular hydrogen bonds are shown as dashed lines.

**Fig. 3.**
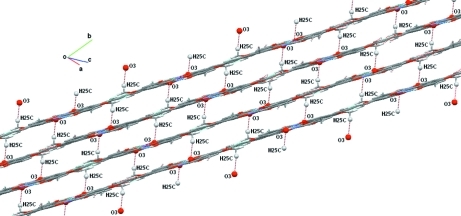
Three-dimensional packing diagram of the title compound, showing the layer configuration along b axis.

**Fig. 4.**
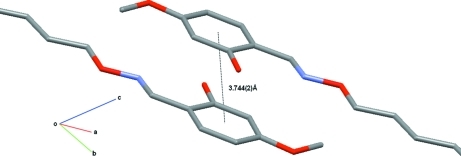
Part π···π stacking interactions in the title compound (as shown as dashed lines). H atoms not involved in hydrogen bonding have been omitted for clarity.

## supplementary crystallographic information

### Comment

Schiff bases are one of most important mixed-donor ligands in the field of modern coordination chemistry. They play an important role in the development of coordination chemistry related to biochemistry, synthesis and catalysis (Mohand *et al.*, 1995; Campbell *et al.*, 2001), enzymatic reactions, magnetism, and supramolecular architectures. Our group are interested in the synthesis and structure of salen-type bisoxime compounds formed by Schiff base reactions (Dong *et al.*, 2008*a*; Dong *et al.*, 2008*b*). Herein, we report, the synthesis and crystal structure of a salen-type bisoxime compound containing nine-methene bridge, 5,5'-dimethoxy-2,2'-[(nonane-1,9-diyldioxy)bis(nitrilomethylidyne)]diphenol.

The molecular structure of the title compound, C_25_H_34_N_2_O_6_, as shown in Fig. 1, adopts a linear configuration, which is similar to our previously reported compound (Sun *et al.*, 2009). In the crystal unit, the oxime (–CH=N—O–) group is coplanar with the aromatic ring and the two benzene rings are approximately parallel, making a dihedral angle of 0.16 (3)°. The bond lengths and angles in the molecule are within normal ranges.

In the crystal structure, strong intramolecular O—H···N hydrogen bonds generate two six-membered rings, producing S(6) ring motifs (Fig. 1) (Bernstein *et al.*, 1995). Intermolecular C—H···O hydrogen bonds (Fig. 2) link the each molecule to five others, foming an infinite three-dimensional supramolecular structure (Desiraju, 1996) (Fig. 3). The crystal is further stabilized by π-π stacking interactions between the neighbouring benzene rings (centroid-centroid distances = 3.744 (2) Å) (Fig. 4).

### Experimental

5,5'-Dimethoxy-2,2'-[(nonane-1,9-diyldioxy)bis(nitrilomethylidyne)]diphenol was synthesized according to an analogous method reported earlier (Dong *et al.*, 2008*c*; Dong *et al.*, 2009). To an ethanol solution (5 ml) of 4-methoxy-2-hydroxybenzaldehyde (159.5 mg, 1.05 mmol) was added an ethanol solution (5 ml) of 1,9-bis(aminooxy)nonane (99.0 mg, 0.52 mmol). The reaction mixture was stirred at 328 K for 8 h. The formed precipitate was separated by filtration under reduced pressure, and washed successively with ethanol and ethanol-hexane (1:4), respectively. The product was dried under vacuum to yield 155.4 mg of the title compound. Yield, 32.3%. m. p. 465–467 K. Anal. Calcd. for C_25_H_34_N_2_O_6_: C, 62.67; H, 6.51; N, 9.96. Found: C, 62.79; H, 6.68; N, 9.83.

Colorless block-like single crystals suitable for X-ray diffraction studies were obtained after about three weeks by slow evaporation from an ethanol solution of the title compound.

### Refinement

Non-H atoms were refined anisotropically. H atoms were treated as riding atoms with distances C—H = 0.96 (CH_3_), C—H = 0.97 (CH_2_), 0.93 Å (CH), O—H = 0.82 Å and *U*_iso_(H)= 1.20 1.2 *U*_eq_(C) for methylene and methylidyne, 1.50 *U*_eq_(C) for methyl, 1.50 *U*_eq_(O) for hydroxy.

### Crystal data

**Table:**

C_25_H_34_N_2_O_6_	*Z* = 2
*M**_r_* = 458.54	*F*(000) = 492
Triclinic, *P*1	*D*_x_ = 1.231 Mg m^−^^3^
Hall symbol: -P 1	Melting point = 465–467 K
*a* = 10.4701 (10) Å	Mo *K*α radiation, λ = 0.71073 Å
*b* = 11.1249 (12) Å	Cell parameters from 1465 reflections
*c* = 12.7602 (14) Å	θ = 2.2–22.6°
α = 65.544 (1)°	µ = 0.09 mm^−^^1^
β = 86.664 (2)°	*T* = 298 K
γ = 67.085 (1)°	Block-like, colorless
*V* = 1236.7 (2) Å^3^	0.48 × 0.46 × 0.21 mm

### Data collection

**Table:**

Bruker SMART 1000 CCD area-detector diffractometer	4303 independent reflections
Radiation source: fine-focus sealed tube	2050 reflections with *I* > 2σ(*I*)
graphite	*R*_int_ = 0.036
φ and ω scans	θ_max_ = 25.0°, θ_min_ = 1.8°
Absorption correction: multi-scan (*SADABS*; Sheldrick, 1996)	*h* = −12→12
*T*_min_ = 0.959, *T*_max_ = 0.982	*k* = −13→13
6463 measured reflections	*l* = −9→15

### Refinement

**Table:**

Refinement on *F*^2^	Primary atom site location: structure-invariant direct methods
Least-squares matrix: full	Secondary atom site location: difference Fourier map
*R*[*F*^2^ > 2σ(*F*^2^)] = 0.049	Hydrogen site location: inferred from neighbouring sites
*wR*(*F*^2^) = 0.125	H-atom parameters constrained
*S* = 1.05	*w* = 1/[σ^2^(*F*_o_^2^) + (0.0355*P*)^2^] where *P* = (*F*_o_^2^ + 2*F*_c_^2^)/3
4303 reflections	(Δ/σ)_max_ < 0.001
300 parameters	Δρ_max_ = 0.16 e Å^−^^3^
0 restraints	Δρ_min_ = −0.18 e Å^−^^3^

### Special details

**Table:**

Geometry. All e.s.d.'s (except the e.s.d. in the dihedral angle between two l.s. planes) are estimated using the full covariance matrix. The cell e.s.d.'s are taken into account individually in the estimation of e.s.d.'s in distances, angles and torsion angles; correlations between e.s.d.'s in cell parameters are only used when they are defined by crystal symmetry. An approximate (isotropic) treatment of cell e.s.d.'s is used for estimating e.s.d.'s involving l.s. planes.
Refinement. Refinement of *F*^2^ against ALL reflections. The weighted *R*-factor *wR* and goodness of fit *S* are based on *F*^2^, conventional *R*-factors *R* are based on *F*, with *F* set to zero for negative *F*^2^. The threshold expression of *F*^2^ > σ(*F*^2^) is used only for calculating *R*-factors(gt) *etc*. and is not relevant to the choice of reflections for refinement. *R*-factors based on *F*^2^ are statistically about twice as large as those based on *F*, and *R*- factors based on ALL data will be even larger.

### Fractional atomic coordinates and isotropic or equivalent isotropic displacement parameters (Å^2^)

**Table:**

	*x*	*y*	*z*	*U*_iso_*/*U*_eq_	
N1	0.2659 (2)	−0.4795 (2)	0.60645 (19)	0.0660 (6)	
N2	0.7314 (2)	0.4634 (2)	0.87062 (18)	0.0634 (6)	
O1	0.34859 (18)	−0.40335 (19)	0.59502 (16)	0.0766 (6)	
O2	0.72925 (18)	0.37983 (19)	0.81319 (15)	0.0766 (6)	
O3	0.07937 (17)	−0.58178 (17)	0.68503 (15)	0.0769 (6)	
H3	0.1269	−0.5362	0.6794	0.115*	
O4	0.02223 (19)	−0.88667 (18)	0.53611 (16)	0.0767 (6)	
O5	0.67807 (19)	0.59846 (18)	1.00861 (15)	0.0830 (6)	
H5	0.6699	0.5472	0.9805	0.124*	
O6	0.9436 (2)	0.87142 (19)	0.98147 (17)	0.0857 (6)	
C1	0.3053 (3)	−0.3278 (3)	0.6680 (2)	0.0719 (8)	
H1A	0.3108	−0.3948	0.7478	0.086*	
H1B	0.2098	−0.2567	0.6422	0.086*	
C2	0.4015 (3)	−0.2568 (3)	0.6585 (2)	0.0746 (8)	
H2A	0.3948	−0.1920	0.5777	0.089*	
H2B	0.4963	−0.3301	0.6813	0.089*	
C3	0.3762 (3)	−0.1723 (3)	0.7304 (2)	0.0681 (8)	
H3A	0.2810	−0.0999	0.7097	0.082*	
H3B	0.3871	−0.2369	0.8119	0.082*	
C4	0.4758 (2)	−0.0993 (3)	0.7122 (2)	0.0659 (7)	
H4A	0.5705	−0.1727	0.7337	0.079*	
H4B	0.4661	−0.0374	0.6302	0.079*	
C5	0.4563 (2)	−0.0103 (2)	0.7788 (2)	0.0656 (7)	
H5A	0.4717	−0.0727	0.8612	0.079*	
H5B	0.3605	0.0606	0.7605	0.079*	
C6	0.5536 (3)	0.0667 (2)	0.7526 (2)	0.0660 (7)	
H6A	0.6491	−0.0045	0.7689	0.079*	
H6B	0.5367	0.1300	0.6703	0.079*	
C7	0.5397 (3)	0.1549 (3)	0.8196 (2)	0.0695 (8)	
H7A	0.5573	0.0922	0.9019	0.083*	
H7B	0.4446	0.2269	0.8032	0.083*	
C8	0.6393 (3)	0.2293 (3)	0.7898 (2)	0.0715 (8)	
H8A	0.7337	0.1568	0.8035	0.086*	
H8B	0.6196	0.2932	0.7076	0.086*	
C9	0.6336 (3)	0.3152 (3)	0.8561 (2)	0.0728 (8)	
H9A	0.5401	0.3885	0.8445	0.087*	
H9B	0.6594	0.2528	0.9384	0.087*	
C10	0.2957 (2)	−0.5469 (3)	0.5419 (2)	0.0622 (7)	
H10	0.3658	−0.5407	0.4943	0.075*	
C11	0.2216 (2)	−0.6325 (2)	0.5424 (2)	0.0525 (6)	
C12	0.1170 (2)	−0.6471 (2)	0.6125 (2)	0.0510 (6)	
C13	0.0470 (2)	−0.7291 (2)	0.6116 (2)	0.0565 (7)	
H13	−0.0242	−0.7359	0.6578	0.068*	
C14	0.0836 (3)	−0.8008 (2)	0.5414 (2)	0.0566 (7)	
C15	0.1875 (3)	−0.7888 (3)	0.4717 (2)	0.0669 (8)	
H15	0.2120	−0.8371	0.4243	0.080*	
C16	0.2542 (3)	−0.7060 (3)	0.4726 (2)	0.0656 (7)	
H16	0.3239	−0.6983	0.4248	0.079*	
C17	−0.0791 (3)	−0.9095 (3)	0.6107 (2)	0.0883 (9)	
H17A	−0.0392	−0.9493	0.6897	0.132*	
H17B	−0.1096	−0.9755	0.6006	0.132*	
H17C	−0.1575	−0.8194	0.5927	0.132*	
C18	0.8248 (3)	0.5120 (2)	0.8369 (2)	0.0580 (7)	
H18	0.8792	0.4871	0.7829	0.070*	
C19	0.8512 (2)	0.6043 (2)	0.8781 (2)	0.0511 (6)	
C20	0.7786 (2)	0.6443 (3)	0.9615 (2)	0.0573 (7)	
C21	0.8074 (3)	0.7331 (3)	0.9981 (2)	0.0632 (7)	
H21	0.7582	0.7594	1.0537	0.076*	
C22	0.9084 (3)	0.7820 (3)	0.9523 (2)	0.0606 (7)	
C23	0.9834 (3)	0.7427 (3)	0.8705 (2)	0.0670 (8)	
H23	1.0532	0.7748	0.8405	0.080*	
C24	0.9532 (3)	0.6557 (2)	0.8348 (2)	0.0617 (7)	
H24	1.0030	0.6301	0.7792	0.074*	
C25	0.8799 (3)	0.9062 (3)	1.0714 (3)	0.1070 (12)	
H25A	0.7806	0.9555	1.0499	0.161*	
H25B	0.9151	0.9673	1.0842	0.161*	
H25C	0.9005	0.8193	1.1412	0.161*	

### Atomic displacement parameters (Å^2^)

**Table:**

	*U*^11^	*U*^22^	*U*^33^	*U*^12^	*U*^13^	*U*^23^
N1	0.0605 (15)	0.0674 (15)	0.0816 (16)	−0.0295 (13)	0.0040 (13)	−0.0379 (14)
N2	0.0647 (15)	0.0654 (14)	0.0738 (15)	−0.0312 (13)	0.0088 (13)	−0.0376 (13)
O1	0.0729 (13)	0.0867 (13)	0.0998 (14)	−0.0434 (11)	0.0196 (11)	−0.0570 (12)
O2	0.0791 (13)	0.0933 (14)	0.0953 (14)	−0.0536 (12)	0.0278 (12)	−0.0592 (12)
O3	0.0862 (13)	0.0910 (13)	0.0914 (13)	−0.0466 (11)	0.0382 (12)	−0.0665 (12)
O4	0.0870 (14)	0.0836 (14)	0.0873 (14)	−0.0448 (12)	0.0190 (12)	−0.0527 (12)
O5	0.0860 (14)	0.1028 (15)	0.0933 (14)	−0.0538 (12)	0.0403 (12)	−0.0610 (12)
O6	0.1094 (16)	0.0787 (13)	0.0937 (15)	−0.0487 (13)	0.0137 (13)	−0.0494 (12)
C1	0.0660 (19)	0.0783 (19)	0.088 (2)	−0.0345 (16)	0.0122 (17)	−0.0460 (18)
C2	0.0654 (19)	0.0794 (19)	0.093 (2)	−0.0348 (17)	0.0070 (17)	−0.0434 (18)
C3	0.0597 (18)	0.0706 (18)	0.084 (2)	−0.0268 (16)	0.0026 (16)	−0.0405 (17)
C4	0.0593 (18)	0.0659 (18)	0.0772 (19)	−0.0237 (15)	−0.0022 (16)	−0.0347 (16)
C5	0.0592 (17)	0.0637 (17)	0.0777 (19)	−0.0250 (15)	0.0034 (15)	−0.0327 (16)
C6	0.0649 (19)	0.0628 (17)	0.0726 (19)	−0.0252 (15)	0.0001 (16)	−0.0303 (16)
C7	0.0700 (19)	0.0682 (18)	0.0774 (19)	−0.0329 (16)	0.0062 (17)	−0.0322 (16)
C8	0.0739 (19)	0.0740 (18)	0.0771 (19)	−0.0367 (17)	0.0091 (16)	−0.0352 (16)
C9	0.075 (2)	0.0714 (19)	0.089 (2)	−0.0382 (17)	0.0162 (18)	−0.0419 (18)
C10	0.0551 (17)	0.0641 (18)	0.0634 (18)	−0.0177 (15)	0.0060 (15)	−0.0289 (16)
C11	0.0466 (16)	0.0540 (16)	0.0557 (17)	−0.0151 (14)	0.0017 (14)	−0.0264 (14)
C12	0.0492 (16)	0.0474 (15)	0.0514 (16)	−0.0089 (13)	0.0013 (14)	−0.0256 (14)
C13	0.0568 (17)	0.0577 (16)	0.0588 (17)	−0.0221 (14)	0.0082 (14)	−0.0292 (15)
C14	0.0582 (17)	0.0511 (16)	0.0594 (18)	−0.0185 (14)	0.0016 (15)	−0.0251 (15)
C15	0.0669 (19)	0.080 (2)	0.0701 (19)	−0.0280 (16)	0.0146 (17)	−0.0486 (17)
C16	0.0576 (18)	0.0809 (19)	0.0643 (18)	−0.0245 (16)	0.0168 (15)	−0.0407 (17)
C17	0.103 (2)	0.091 (2)	0.095 (2)	−0.059 (2)	0.025 (2)	−0.0444 (19)
C18	0.0515 (17)	0.0588 (17)	0.0602 (17)	−0.0166 (14)	0.0030 (15)	−0.0268 (14)
C19	0.0462 (15)	0.0476 (15)	0.0546 (16)	−0.0142 (13)	0.0002 (14)	−0.0208 (14)
C20	0.0490 (16)	0.0572 (16)	0.0580 (17)	−0.0191 (14)	0.0094 (15)	−0.0201 (15)
C21	0.074 (2)	0.0568 (17)	0.0601 (17)	−0.0202 (16)	0.0069 (16)	−0.0315 (15)
C22	0.0676 (19)	0.0467 (16)	0.0613 (18)	−0.0192 (15)	−0.0061 (16)	−0.0192 (15)
C23	0.0651 (18)	0.0658 (18)	0.077 (2)	−0.0300 (16)	0.0125 (17)	−0.0332 (17)
C24	0.0611 (18)	0.0617 (17)	0.0643 (17)	−0.0230 (15)	0.0104 (15)	−0.0307 (15)
C25	0.161 (3)	0.102 (2)	0.091 (2)	−0.063 (2)	0.028 (2)	−0.064 (2)

### Geometric parameters (Å, °)

**Table:**

N1—C10	1.280 (3)	C7—H7B	0.9700
N1—O1	1.394 (2)	C8—C9	1.501 (3)
N2—C18	1.266 (3)	C8—H8A	0.9700
N2—O2	1.409 (2)	C8—H8B	0.9700
O1—C1	1.443 (3)	C9—H9A	0.9700
O2—C9	1.410 (3)	C9—H9B	0.9700
O3—C12	1.353 (2)	C10—C11	1.441 (3)
O3—H3	0.8200	C10—H10	0.9300
O4—C14	1.366 (3)	C11—C16	1.390 (3)
O4—C17	1.410 (3)	C11—C12	1.390 (3)
O5—C20	1.349 (3)	C12—C13	1.379 (3)
O5—H5	0.8200	C13—C14	1.377 (3)
O6—C22	1.369 (3)	C13—H13	0.9300
O6—C25	1.410 (3)	C14—C15	1.377 (3)
C1—C2	1.476 (3)	C15—C16	1.358 (3)
C1—H1A	0.9700	C15—H15	0.9300
C1—H1B	0.9700	C16—H16	0.9300
C2—C3	1.513 (3)	C17—H17A	0.9600
C2—H2A	0.9700	C17—H17B	0.9600
C2—H2B	0.9700	C17—H17C	0.9600
C3—C4	1.509 (3)	C18—C19	1.446 (3)
C3—H3A	0.9700	C18—H18	0.9300
C3—H3B	0.9700	C19—C24	1.385 (3)
C4—C5	1.504 (3)	C19—C20	1.394 (3)
C4—H4A	0.9700	C20—C21	1.384 (3)
C4—H4B	0.9700	C21—C22	1.367 (3)
C5—C6	1.512 (3)	C21—H21	0.9300
C5—H5A	0.9700	C22—C23	1.383 (3)
C5—H5B	0.9700	C23—C24	1.366 (3)
C6—C7	1.511 (3)	C23—H23	0.9300
C6—H6A	0.9700	C24—H24	0.9300
C6—H6B	0.9700	C25—H25A	0.9600
C7—C8	1.512 (3)	C25—H25B	0.9600
C7—H7A	0.9700	C25—H25C	0.9600
			
C10—N1—O1	112.5 (2)	C8—C9—H9A	110.4
C18—N2—O2	109.9 (2)	O2—C9—H9B	110.4
N1—O1—C1	108.68 (18)	C8—C9—H9B	110.4
N2—O2—C9	110.86 (18)	H9A—C9—H9B	108.6
C12—O3—H3	109.5	N1—C10—C11	120.6 (2)
C14—O4—C17	118.41 (19)	N1—C10—H10	119.7
C20—O5—H5	109.5	C11—C10—H10	119.7
C22—O6—C25	117.9 (2)	C16—C11—C12	117.1 (2)
O1—C1—C2	106.5 (2)	C16—C11—C10	120.6 (2)
O1—C1—H1A	110.4	C12—C11—C10	122.3 (2)
C2—C1—H1A	110.4	O3—C12—C13	116.9 (2)
O1—C1—H1B	110.4	O3—C12—C11	121.6 (2)
C2—C1—H1B	110.4	C13—C12—C11	121.5 (2)
H1A—C1—H1B	108.6	C14—C13—C12	119.3 (2)
C1—C2—C3	115.4 (2)	C14—C13—H13	120.4
C1—C2—H2A	108.4	C12—C13—H13	120.4
C3—C2—H2A	108.4	O4—C14—C15	116.1 (2)
C1—C2—H2B	108.4	O4—C14—C13	123.6 (2)
C3—C2—H2B	108.4	C15—C14—C13	120.3 (2)
H2A—C2—H2B	107.5	C16—C15—C14	119.7 (2)
C4—C3—C2	111.8 (2)	C16—C15—H15	120.2
C4—C3—H3A	109.3	C14—C15—H15	120.2
C2—C3—H3A	109.3	C15—C16—C11	122.2 (2)
C4—C3—H3B	109.3	C15—C16—H16	118.9
C2—C3—H3B	109.3	C11—C16—H16	118.9
H3A—C3—H3B	107.9	O4—C17—H17A	109.5
C5—C4—C3	115.4 (2)	O4—C17—H17B	109.5
C5—C4—H4A	108.4	H17A—C17—H17B	109.5
C3—C4—H4A	108.4	O4—C17—H17C	109.5
C5—C4—H4B	108.4	H17A—C17—H17C	109.5
C3—C4—H4B	108.4	H17B—C17—H17C	109.5
H4A—C4—H4B	107.5	N2—C18—C19	123.3 (2)
C4—C5—C6	113.3 (2)	N2—C18—H18	118.3
C4—C5—H5A	108.9	C19—C18—H18	118.3
C6—C5—H5A	108.9	C24—C19—C20	117.4 (2)
C4—C5—H5B	108.9	C24—C19—C18	119.8 (2)
C6—C5—H5B	108.9	C20—C19—C18	122.8 (2)
H5A—C5—H5B	107.7	O5—C20—C21	118.0 (2)
C7—C6—C5	115.0 (2)	O5—C20—C19	121.3 (2)
C7—C6—H6A	108.5	C21—C20—C19	120.8 (2)
C5—C6—H6A	108.5	C22—C21—C20	119.7 (2)
C7—C6—H6B	108.5	C22—C21—H21	120.1
C5—C6—H6B	108.5	C20—C21—H21	120.1
H6A—C6—H6B	107.5	C21—C22—O6	124.4 (3)
C6—C7—C8	112.7 (2)	C21—C22—C23	120.8 (2)
C6—C7—H7A	109.1	O6—C22—C23	114.8 (3)
C8—C7—H7A	109.1	C24—C23—C22	118.7 (3)
C6—C7—H7B	109.1	C24—C23—H23	120.7
C8—C7—H7B	109.1	C22—C23—H23	120.7
H7A—C7—H7B	107.8	C23—C24—C19	122.5 (2)
C9—C8—C7	115.3 (2)	C23—C24—H24	118.7
C9—C8—H8A	108.4	C19—C24—H24	118.7
C7—C8—H8A	108.4	O6—C25—H25A	109.5
C9—C8—H8B	108.4	O6—C25—H25B	109.5
C7—C8—H8B	108.4	H25A—C25—H25B	109.5
H8A—C8—H8B	107.5	O6—C25—H25C	109.5
O2—C9—C8	106.6 (2)	H25A—C25—H25C	109.5
O2—C9—H9A	110.4	H25B—C25—H25C	109.5
			
C10—N1—O1—C1	178.4 (2)	C12—C13—C14—C15	−1.0 (4)
C18—N2—O2—C9	175.3 (2)	O4—C14—C15—C16	−179.6 (2)
N1—O1—C1—C2	176.5 (2)	C13—C14—C15—C16	0.1 (4)
O1—C1—C2—C3	−179.1 (2)	C14—C15—C16—C11	0.3 (4)
C1—C2—C3—C4	−178.0 (2)	C12—C11—C16—C15	0.1 (4)
C2—C3—C4—C5	178.9 (2)	C10—C11—C16—C15	179.3 (2)
C3—C4—C5—C6	−176.9 (2)	O2—N2—C18—C19	178.71 (19)
C4—C5—C6—C7	−178.7 (2)	N2—C18—C19—C24	−178.5 (2)
C5—C6—C7—C8	−180.0 (2)	N2—C18—C19—C20	2.1 (4)
C6—C7—C8—C9	−178.3 (2)	C24—C19—C20—O5	−179.7 (2)
N2—O2—C9—C8	−179.98 (18)	C18—C19—C20—O5	−0.2 (3)
C7—C8—C9—O2	−177.8 (2)	C24—C19—C20—C21	0.6 (3)
O1—N1—C10—C11	179.65 (19)	C18—C19—C20—C21	−179.9 (2)
N1—C10—C11—C16	−179.8 (2)	O5—C20—C21—C22	−179.9 (2)
N1—C10—C11—C12	−0.7 (4)	C19—C20—C21—C22	−0.2 (4)
C16—C11—C12—O3	178.9 (2)	C20—C21—C22—O6	179.2 (2)
C10—C11—C12—O3	−0.3 (3)	C20—C21—C22—C23	−0.7 (4)
C16—C11—C12—C13	−1.0 (3)	C25—O6—C22—C21	5.4 (4)
C10—C11—C12—C13	179.8 (2)	C25—O6—C22—C23	−174.7 (2)
O3—C12—C13—C14	−178.4 (2)	C21—C22—C23—C24	1.1 (4)
C11—C12—C13—C14	1.5 (3)	O6—C22—C23—C24	−178.8 (2)
C17—O4—C14—C15	176.3 (2)	C22—C23—C24—C19	−0.6 (4)
C17—O4—C14—C13	−3.4 (3)	C20—C19—C24—C23	−0.2 (3)
C12—C13—C14—O4	178.7 (2)	C18—C19—C24—C23	−179.7 (2)

### Hydrogen-bond geometry (Å, °)

**Table:**

*D*—H···*A*	*D*—H	H···*A*	*D*···*A*	*D*—H···*A*
C17—H17B···O4^i^	0.96	2.68	3.369 (3)	129
C18—H18···O3^ii^	0.93	2.40	3.295 (3)	160
C25—H25C···O3^iii^	0.96	2.59	3.541 (4)	170
C25—H25B···O6^iv^	0.96	2.61	3.469 (4)	150
O3—H3···N1	0.82	1.86	2.583 (4)	147
O5—H5···N2	0.82	1.94	2.663 (4)	147

Symmetry codes: (i) −*x*, −*y*−2, −*z*+1; (ii) *x*+1, *y*+1, *z*; (iii) −*x*+1, −*y*, −*z*+2; (iv) −*x*+2, −*y*+2, −*z*+2.
